# Prior trauma experiences among state patients charged with murder: A retrospective records review

**DOI:** 10.4102/sajpsychiatry.v30i0.2190

**Published:** 2024-03-01

**Authors:** Ugasvaree Subramaney, Nashia Minty, Chad M. Himlok, Damilola Adetiba, Hamza Ahmed, Elouise Barnard, Yolanda R. Mahachi, Koketso Selekana, Jenna R. Smith, Samantha Iyaloo

**Affiliations:** 1 Department of Psychiatry, School of Clinical Medicine, Faculty of Health Sciences, University of the Witwatersrand, Johannesburg, South Africa; 2 School of Clinical Medicine, Faculty of Health Sciences, University of the Witwatersrand, Johannesburg, South Africa; 3 Research and Policy Unit, Council for Medical Schemes, Pretoria, South Africa

**Keywords:** state patients, trauma, criminal procedures act, violence, mental illness

## Abstract

**Background:**

Trauma experiences, particularly in childhood, have been associated with criminality and mental illness. There is a paucity of research into the crime of murder, trauma and mental illness.

**Aim:**

This research study focused on state patients charged with murder and sought to determine associations with prior trauma experiences, and specific types of traumas (sexual, physical and emotional).

**Setting:**

The study was conducted at a forensic psychiatric hospital in Johannesburg, South Africa.

**Methods:**

The records of state patients admitted over a 21-year period on a charge of murder were reviewed and analysed with respect to sociodemographic variables, clinical profiles, trauma experience and victim characteristics.

**Results:**

Experience of trauma in this population, with a much higher number of males compared with females, was lower than previous studies. Neurocognitive disorder was significantly associated with physical trauma. Physical trauma was found to have a significantly negative association with psychotic disorders, compared with other mental disorders.

**Conclusion:**

Although trauma is common in psychiatric patients, the study’s findings are lower than previous studies. Noting the male bias in state patients that commit murder, greater sample sizes are needed to adequately address issues of specific types of traumas, the development of mental illness and murder.

**Contribution:**

Exploring and managing prior trauma in state patients who commit murder is important while considering rehabilitation efforts, such that reintegration into the community and non-recidivism are encouraged.

## Introduction

South Africa (SA) has a high rate of violence and abuse, evidenced by its inclusion in the country’s quadruple disease burden (together with HIV, maternal and child health and noncommunicable diseases).^1^ South Africa also has the third highest number of murders per population in the world.^2^ Physical, sexual and emotional abuse (henceforth, collectively referred to as ‘trauma’) has a profound, detrimental impact on an individual’s psyche. Radmanovic found that victims of childhood sexual abuse subsequently developed depression, anxiety, post-traumatic stress disorder (PTSD) and other severe mental illnesses, such as schizophrenia, especially in those already predisposed to mental illness.^3^ Furthermore, the risk for violent and antagonistic behaviour is closely associated with a history of exposure to traumatic abuse.^4^ Therefore, there is a relationship between trauma exposure and mental illness as well as a relationship between trauma exposure and violence or violent offenses. Additionally, mental illness has been linked to more extreme forms of violence, specifically murder.^5^ A narrative review by Almomen et al. found a lack of evidence linking mental illness independently with homicide, both globally and in Arab countries.^6^ However, there remains a paucity of research on the intersection between the three elements – trauma exposure, mental illness and homicide. This retrospective review thus aims to investigate the prevalence of exposure to trauma among state patients (those who commit serious or violent offences and are found not fit and/or not responsible by virtue of mental illness)^7^ charged with the crime of murder. In addition, as most literature on abuse focuses on the prevalence, experience and consequences on children and adult women, the dearth of literature on adult males’ experience of trauma is a further motivation for this study.

## Background

In this article, ‘trauma’ will be used as an umbrella term to encompass the exposure to physical, sexual and/or emotional abuse.

South Africa has a high burden of all three types of traumatic experiences. One study on child abuse in SA found that the prevalence of sexual, physical and emotional abuse in a sample of school-going children (*n* = 5635) was 19.8%, 34.4% and 16.1%, respectively.^8^ Another study found that one in four women experience abuse across their lifetime.^9^ In an unpublished Master of Medicine research report, Eklektos observed that these statistics allow for an appreciation of the pervasiveness of abuse in SA and give an idea of the rates one might expect to find in the mentally ill population who, like children, are deemed a vulnerable group.^10^

Regarding the degree of trauma exposure in the mentally ill population specifically, there is little SA literature and an appreciable dearth worldwide. A study performed in India found the prevalence of abuse to be 72% in a population of mentally ill patients. Of these, 39% experienced physical abuse, 21% sexual abuse and 64% emotional abuse.^11^ The study found a correlation between abuse and certain psychiatric disorders. Another study, conducted in Norway,^12^ found the rate of trauma exposure among psychiatric patients to be as high as 91%, stating that trauma exposure is higher among this group compared with the general population. This is because mental illness is a risk factor for experiencing trauma and trauma is a risk factor for developing mental illness.^12^ This bidirectional relationship between trauma and mental illness is complex and not linear, making it difficult to determine cause and effect.

Victims of abuse tend to have experienced more than one of the three forms – making it difficult to assess the specific impact of each type of trauma on an individual’s mental health.^13^ Despite this, correlations have been drawn between different types of trauma experiences and the development of any particular mental illnesses. Adults who experienced childhood sexual abuse have an increased risk of presenting with PTSD, schizophrenia and substance use disorders (SUDs).^14^ Physical abuse was associated with an increased risk of PTSD and panic disorder. In addition, these victims were twice as likely to develop childhood behavioural and conduct disorders.^14^ Adults who experienced emotional abuse in childhood were more likely to develop depression, anxiety, stress and a neurotic personality. Emotional abuse has been found to have more detrimental developmental consequences than other forms of abuse.^15^

Publications on the physiological effects of trauma on neurological pathways and brain development indicate that exposure to trauma, especially among men, is a major predictor for violent and criminal tendencies.^4^ A proposed reason for the development of this antisocial demeanour is the biological impact that trauma has on certain neurological structures – the corpus callosum and amygdala specifically.^16^ Emotional and sexual abuse leads to a stunted development of certain regions in the corpus callosum. It is further suggested that because of this reduction, an individual may struggle to inhibit rage or use logic, which provokes emotional dysregulation. Development of the amygdala is also impaired, which is associated with raised hostility.^17^ These data suggest that trauma is intimately related to violence and neurological pathology.

Regarding the crime of murder, there is a notable close association with severe mental illness. A study conducted in Australia showed that the rise in the murder rate was directly proportional to the rise in the number of convicted murderers presenting with schizophrenia.^18^ Furthermore, patients with personality disorders such as antisocial personality disorder (ASPD) and paranoid personality disorder were markedly more inclined to violent behaviour and the probability of committing homicide.^19^ Personality disorders were also found to be the most common primary diagnosis of homicide offenders.^19^ A study of 29 convicted murderers/attempted murderers in Brazil indicated that 51.72% of them were diagnosed with ASPD and a 68% prevalence of substance abuse among those convicted for violent crimes, including murder.^5^

Trauma is therefore rife, with physical, sexual and emotional abuse shown to have numerous long-term effects including many severe mental illnesses (such as schizophrenia, ASPD and SUD), which have been found to have a high incidence among mentally ill murderers. In particular, childhood trauma has been shown to impair the development of various neurological structures, leading to hostile behaviour, especially among adult men.

This study hypothesised that a history of trauma will be common (>50%) among mental health care users (MHCUs) charged with and found not fit or not criminally responsible for the crime of murder. We also postulated that female state patients will have experienced more trauma than males.

This study had the following objectives:

- To ascertain the demographic and clinical profile of mentally ill persons who have committed murder
- To determine the relationship between particular types of traumas and certain mental illnesses
- To determine the most prevalent type of trauma among MHCUs charged with murder
- To evaluate offence characteristics such as the relationship of victims with the perpetrator and the presence of substance use at the time.
- To demonstrate the proportion of patients receiving and compliant with treatment for their mental illness at the time of the murder.

## Research methods and design

### Study design

This study is a retrospective records review of forensic state patients, who committed the crime of murder. The period of the review (2000–2020) was 21 years. This ensured an adequate sample size.

### Study site

The forensic unit of Sterkfontein Hospital in Johannesburg, SA.

### Sample population

The records of 152 forensic state patients over the age of 18 who were charged with the crime of murder, admitted from 01 January 2000 to 31 December 2020, were reviewed. After a 30-day observation period at a forensic psychiatric facility, psychiatrists make a recommendation for the management of the individual as a state patient, which usually entails medication and rehabilitation.

### Data collection methods

Once approval was obtained from the Chief Executive Officer (CEO) of the psychiatric hospital and the University of the Witwatersrand Human Research and Ethics Committee (HREC), the first author retrieved the files from the hospital registry and, in accordance with the study sample criteria, made copies of the forensic patient records (specifically the final mental report and the psychiatric report in terms of the Criminal Procedure Act [CPA]).^20^ All identifiers were removed and the data collectors (medical students in their 5th year of study) reviewed the records. The data were recorded onto a Microsoft Excel data sheet incorporating the following: sociodemographic details, diagnosed mental illness, previous trauma experience (including nature, age at which trauma was experienced), victim characteristics including relationship to the perpetrator, age of the MHCU at the time of the offence, clinical treatment at the time, if appropriate and use of alcohol or illicit substances at the time of the offence.

### Ethical considerations

Only the records of state patients were reviewed. There were no patients admitted at the hospital at the time of the research study. The University of the Witwatersrand Human Ethics and Research Committee approved the research project. (study number M210498) as well as approval from the hospital management was obtained. The study was registered on the National Health Research Database (number 202110010). Unique identifiers were allocated to patients in the data sheet as opposed to using any personal details (e.g. name, surname, date of birth) to maintain patients’ confidentiality. Only the researchers had access to the captured data. While consent is not usually sought in a retrospective record review, if any records belonged to state patients still hospitalised at the time of the data collection, consent from these patients to review their records was obtained by the first author, in keeping with the hospital’s protocol. Suitability to participate, that is, capacity to consent was obtained from treating consultant of the ward where appropriate. In the event that any state patient became distressed because of the nature of the research, they were referred to the treating doctor and psychologist of the ward.

### Data analysis

Data were analysed using StataSE version 17.0 (StataCorp. 2021, Stata Statistical software:Release 17. College Station,TX: StataCorp LLC.). Chi-square tests were used to test for associations between variables, taking a *p* < 0.05 as the level of significance. Simple logistic regression was used to test the strength of the associations between variables using odds ratio and 95% confidence interval (CI). The following associations were assessed:

- Types of trauma and certain mental illnesses
- Trauma prevalence in males and females
- Victim characteristics in males and females

## Results

### Sociodemographic profile

Table 1 summarises the sociodemographics of the study population. From a sample of 152 adult forensic state patients 117 (77%) patients were male and 35 (23%) were female. Sixty-eight (45%) were in the age range of 31–50. The majority (94, 62%) of patients were unemployed.

**TABLE 1:** Sociodemographic characteristics in the sample population.

Variable	*N*	%
**Sample size**	**152**	100
Male: Female	117:35	77:23
**Age†**		
18–30	66	43
31–50	68	45
> 50	17	11
**Employment**		
Employed	41	27
Unemployed	94	62
Piece jobs	7	27
Retired	2	1
Unknown	8	5

† , Age range: 18–67.

### Substance use

Substance use was present in 75 (49%) patients with 23 (15%) using alcohol alone, 20 (13%) using alcohol and cannabis, and 12 (8%) using alcohol and other substances. A total of 73 (48%) patients were not using any substances at the time of offence. Many were using more than one substance. Ten patients (7%) were on stimulants; four abused cocaine, three ecstasy, one amphetamines, one methamphetamines and one unspecified stimulants. Fifteen patients (10%) were using alcohol in conjunction with multiple other substances including mandrax, glue, inhalants, nyaope, lysergic acid diethylamide (LSD), heroin and stimulants. Two (1%) patients were using a combination of alcohol, cannabis and mandrax. One patient was using only nyaope, and one patient was using only cocaine. In one patient, substance use was confirmed but the exact substances used were unknown. In 3% of cases, substance use was unknown.

### Victims’ characteristics

The number of patients who murdered first-degree relatives, friends, strangers, acquaintances, other relatives and others is depicted in Table 2. Females were more likely to murder a first-degree relative.

**TABLE 2:** Victims’ characteristics by gender.

Victims	*N*	%
**Sample size**	**152**	-
**Overall**		
First-degree relatives	55	36
Strangers	45	30
Other relatives	22	14
Acquaintances	15	10
Friends	10	7
Unknown	3	3
**Female**		
First-degree relatives	23	66
Other relatives	7	20
Strangers	3	8
Acquaintances	1	3
**Male**		
Strangers	38	34
First-degree relatives	32	28
Acquaintances	21	19
Other relatives	12	11
Friends	9	8

### History of mental illness and clinical diagnoses

Fifty-five (36%) patients had a previous diagnosis of mental illness and only 21 (38%) of these were on treatment at the time of the murder. For the remaining 97 (64%) patients, it was the index 103 episode.

Noting the presence of comorbid conditions, 108 (71%) patients were diagnosed with a psychotic disorder, 27 (18%) with a neurocognitive disorder, 21 (14%) with SUD, 12 (8%) with neurodevelopmental disorder, 11 (7%) with mood disorder, three (2%) with substance induced psychotic disorder, two (1%) with personality disorders and only one (<1%) with PTSD.

### Trauma

A total of 62 (41%) patients experienced trauma. In 6% of cases, it was unknown.

Some patients experienced a combination of traumas. Seven patients (11%) experienced trauma at an unknown age, 25 (38%) patients experienced trauma in childhood and 30 (46%) in adulthood. Three (5%) of patients experienced trauma in both childhood and adulthood. As a result of significantly lower number of females, a sex comparison for types of traumas experienced was not carried out.

### Statistical analyses

A greater number of MHCUs did not experience trauma, compared with those who did (*p* = 0.047). The presence of trauma did not vary significantly between male and females (*p* = 0.990). Females were more likely to murder a first-degree relative than males (*p* < 0.05).

Patients who had been diagnosed with psychotic disorder were more likely to have experienced emotional trauma (odds ratio [OR] = 0.31, *p* < 0.86, CI [0.15–0.66]), than sexual trauma (OR = 0.63, *p* < 0.4) and physical trauma (OR = 0.31, *p* < 0.002). These patients were also 37% less likely to have experienced sexual trauma than those without trauma and 69% less likely to have experienced physical trauma than those without trauma (*p* = 0.00; 95% CI [0.15–0.66]).

Patients with neurocognitive disorder were 4.36 times more likely to have physical trauma compared with those without trauma (*p* = 0.001, CI [1.81–10.51]). Other insignificant associations are that participants with neurocognitive disorder were 62% less likely to have sexual trauma compared with those with no trauma (*p* = 0.36). Patients with neurocognitive disorder were 85% less likely to have emotional trauma compared with those with no trauma (*p* = 0.26).

Insignificant associations were found between neurocognitive disorder and sexual trauma and neurocognitive disorder and emotional trauma. The relationship between SUD and different traumas were all insignificant (see Table 4).

**TABLE 3:** Types of traumas experienced.

Trauma experienced	*N*	%
Physical	45	70
Emotional	23	36
Sexual	13	20
Uncertain	6	4

**TABLE 4:** Association between mental illness and trauma exposure.

Mental illness	Physical trauma (*n* = 45)	Sexual trauma (*n* = 13)	Emotional trauma (*n* = 23)
Psychotic disorder (*n* = 87)	Odds ratio 0.31*	Odds ratio 0.63	Odds ratio 0.91
Neurocognitive disorder (*n* = 26)	Odds ratio 4.36*	Odds ratio 0.38	Odds ratio 0.42
Substance use disorder (*n* = 21)	Odds ratio 2.49	Odds ratio 0.5	Odds ratio 0.93

* , *p* ≤ 0.05.

## Discussion

### Demographics

As was anticipated, more male patients committed murder than females. This is slightly less than the 87% male portion shown in a previous study at Sterkfontein Hospital in SA on forensic patients^21^ and similar to the retrospective review of patients by Hayes et al.^22^

The mean age of 38 years old is in keeping with Sterkfontein Hospital studies^10,21^ (mean ages 32 and 38 years old, respectively). An Australian study also found the mean age among dual diagnosis forensic psychiatric patients to be 40 years old.^23^ Previous studies noted much higher unemployment rates in forensic populations (88.5% and 78%)^9,20^ compared with our study.^22^

### Prevalence of trauma

Despite the well-established South African burden of trauma and abuse, both hypotheses set out were not proven. Less than half of the sample population experienced trauma and females did not experience a significantly higher rate of trauma than males. Previous literature demonstrated a strong correlation between mental illness and lifetime prevalence of experiencing trauma, with concordance rates of 70% – 90%.^13^ Furthermore, while females experienced a higher rate of trauma than the general population of women,^10^ it was still lower than expected given the levels of gender-based violence in South Africa compounded with the trauma rates among mentally ill individuals. The limitation of the marked male-to-female ratio of distribution of the population sample accounts for this.

The discrepancy between the current and previous findings might be because of the limitations of record reviews, resulting in differences in the way histories are shared with and captured by different clinicians; as well as discomfort by patients to share all the details about their trauma experiences at the time of admission. Nevertheless, there was still a high percentage of patients who reported a history of trauma. This serves as a reminder to sensitively ask about trauma experiences while performing a psychiatric interview with state patients in order to aid in rehabilitation efforts.

### Distribution of trauma types

The rate of physical trauma in this study was lower than previously found by Artz et al.^8^ (34.4%) and Eklektos (39.2%).^10^ Similarly, history of sexual trauma was markedly lower than in the two above-mentioned studies (9% vs. 19.4% and 24.6%, respectively). Lastly, 15.1% had experienced emotional trauma, which is in keeping with Artz et al.^8^ (16.1%), but much lower than the 44.6% found by Eklektos.^10^

### Mental illness correlations

Most patients had some form of psychotic disorder, mirroring Valenca and de Moraes’s findings, where patients with schizophrenia were more inclined towards violent behaviour.^5^ This relationship between psychotic disorders and homicide has previously been demonstrated as a direct relationship between the rise in murder rates and number of perpetrators diagnosed with schizophrenia.^19^

Physical trauma was found to have a significantly negative association with psychotic disorders, as compared with other forms of mental illness. The significantly strong positive association between physical trauma and neurocognitive disorder is in keeping with the impaired development of brain structures following childhood physical trauma.^15^

### Offence characteristics

While exploring the relationship with the victims, females murdered first-degree relatives by nearly double the rate compared with their male counterparts. This is further corroborated by a study researching women in forensic mental health units in South Africa, wherein the victims were typically immediate family and their direct inner social circle.^24^

### History of mental illness

Just over a third of the sample population presented with their index episode at the time of offence, a significantly lower proportion of patients than in Nagdee’s study, where 51% had no prior psychiatric history.^24^ The difference in sex between the two studies may account for this difference, with the present study consisting of a majority male sample. Furthermore, only a minority of patients with a prior diagnosis were compliant with treatment at the time of the murder. This poor pattern of adherence lends scope for future research into pharmacological interventions and compliance with patients known with psychiatric illness, especially among those with risk factors for violent tendencies.

### Limitations

The findings of this study are limited to the South African context as only patients from a single hospital and single setting were reviewed. Furthermore, the sample size was too small to conclude significant relationships between certain variables. The recorded information is reliant upon the accuracy with which it has been obtained and captured by different clinicians. Issues may have been encountered while reporting this information because of factors such as substance use and trauma as well as the fact that many MHCUs provide inaccurate information as a result of psychopathology. Incomplete and/or inaccurate patient records as well as lost and/or missing patient records are further limitations. The study did not consider possible confounders and effect modifiers that could have altered the relationship between trauma and mental illness such as socio-economic status, education, support structure, etc. Multivariate analyses were also not performed.

Furthermore, the period of the review was 21 years, and the clinical diagnoses of mental disorders were subject to the Diagnostic and Statistical Manual of Mental Disorders (DSM) edition in use at the time.

## Conclusion and recommendations

This study found that while trauma exposure among mental healthcare users charged with the crime of murder was high, it was lower than other studies. A significant positive correlation was found between physical trauma and neurocognitive disorders. Sex specific associations could not be ascertained. It is recommended that mediating and moderating variables should be examined using a larger sample size, in a cross-sectional design.
